# Novel Electrodes and Engineered Interfaces for Halide-Semiconductor Radiation Detectors

**DOI:** 10.1038/s41598-019-46360-z

**Published:** 2019-07-09

**Authors:** Amlan Datta, Piotr Becla, Shariar Motakef

**Affiliations:** CapeSym, Inc., Natick, MA 01760 USA

**Keywords:** Applied physics, Materials for devices

## Abstract

A new class of inorganic halide semiconductors are emerging as high-efficiency low-cost candidates for spectroscopic radiation detection. We report on solving one of the major challenges of these halide radiation detectors. At room temperature halide semiconductor detectors polarize under applied electric field, which not only degrades the charge collection efficiency of the detectors, but also promotes chemical reaction of the metal electrodes with the halide ions. This increases the metal-semiconductor interface noise and early failure of the spectroscopic detection capabilities of the device. We report on a solution to this challenge by application of novel electrodes on Thallium Bromide (TlBr) radiation detectors with virtually defect-free electrode-semiconductor interfaces, showing low noise and high detection stability for an extended period of time under accelerated ageing conditions. A number of TlBr detectors fabricated by this technique have demonstrated continuous stable detection performance (e.g. ±1% change in 662 keV gamma channel) for more than 4000 hours at room temperature. This report also shows continuously recorded ^137^Cs gamma radiation response of a unidirectionally-biased pixelated TlBr detector over more than 2 months (a total of 2880 data sets), which exhibit excellent stability. The developed approach has resulted in unprecedented low-noise stable performance of halide semiconductor detectors at room temperature, overcoming one of the major obstacles to the full consideration of TlBr (and other halide semiconductors) as a potentially low-cost replacement for Cadmium Zinc Telluride (CZT).

## Introduction

A new class of solid-state ionizing radiation detectors are emerging with the advent of Thallium Bromide (TlBr) and perovskite halide crystals such as CsPbBr_3_ that have been demonstrated to be excellent γ-detectors^1^. However, the deleterious effects of electromigration of the halide, in particular Br-ions, during the room temperature operation of these detectors has inhibited their adoption for use in non-laboratory radiation detection instruments^2,3^. In this paper we demonstrate a successful approach to addressing this problem, which will elevate TlBr, and possibly other halide semiconductors, to serious alternatives to CZT.

At present CZT detectors are the only room temperature uncooled semiconductor detectors with very high energy resolution. However, CZT detectors continue to be very expensive, primarily due to the low yield of the crystal growth process. In spite of significant progress in growth of high purity crystals by the Travelling Heater Method (THM), the innate properties of CZT lead to compositional non-uniformities and crystalline defects that limit availability of material for high resolution spectral detectors. Investigations to improve the crystal growth yield through replacing Zn with other metals such as Mn, Mg and Se, have met limited success to date^4^. Compared to CZT, the halide semiconductor TlBr offers several distinct advantages. Due to the presence of heavy Tl metal, TlBr has a larger absorption coefficient, resulting in approximately 50% higher radiation attenuation as compared to CZT at 662 keV^5^. The high electrical resistivity (>10^10^ Ohm-cm) of TlBr is achieved by self-compensation. Therefore, TlBr crystals do not experience the compositional variations related to the distribution of the dopant as seen in In-doped CZT. The lower growth temperature of TlBr (480 °C) significantly reduces the complexity of the growth and purification equipment. Also, as TlBr has a cubic crystal structure, melt-based crystal growth techniques produce single crystalline boules, whereas production of single crystal boules is a significant challenge for CZT. TlBr detectors fabricated from high purity and stoichiometric TlBr crystals have high mobility-lifetime product values of up to 10^−2^ cm^2^/V for electrons, similar to high quality CZT detectors. As the mobility of holes and electrons in TlBr are of the same order (unlike CZT), planar detectors with 662 keV energy resolution of 2.5% (without digital correction) can be routinely produced.

Halide semiconductors are ionic in nature, and under an applied electric field, the positive (Tl^+^) and negative (Br^−^) ions tend to electro-diffuse^6–11^ through vacancy-hopping^12^. The stability of TlBr radiation detectors is primarily influenced by the faster electro-diffusion of the Br-ions towards the anode, and to a lesser extent Tl^+^ towards cathode. Br-ions react aggressively with nearly all types of metallic anodes, and form non-conducting metal bromides (e.g., AuBr_3_, PtBr_2_). As a result, the electric field near the electrode region changes resulting in high detector noise as well as progressive deterioration of charge collection efficiency of the detector. The noise associated with high frequency changes in the electric field has been reported for other ionic halide semiconductors (e.g., HgI_2_)^13^. We have also observed this phenomenon’s manifestation through Pockels effect experiments^14^. The chemical reaction occurring at the anode is the primary cause of long-term device failure and continuous increase in baseline noise^8,9,15^.

Several approaches to achieving long lifetime in TlBr detectors have been investigated. Operation of these devices at sub-zero temperatures (to about −20 °C) where both the metal-bromine formation energy and the diffusivity of ions are appreciably reduced have been successful, but complications associated with cooling of the devices is a major impediment to their use in detection instruments^16^. Other published approaches have focused on eliminating the reaction of Br-ions with the electrode, such as creation of TlBr_1-x_Cl_x_ heterojunction at the metal-TlBr interface^15^ and use of various metals as electrodes including Pt, Au, Pd and Tl^7,17^. However, these approaches have not been fully successful, and the maximum room temperature lifetime has been less than 2000 hours at relatively moderate electric field strength of 1000 V/cm. More importantly, the results are not reproducible, and both the device performance and lifetime vary significantly from one detector to the other. A successful approach has been periodic switching of the bias^18^. A stable operational lifetime of five years at 8 hours/day was obtained for planar and pixelated detectors. The optimal switching period has been found to be about 8 hours with the maximum period being 24 hours^18^.

In this paper, we report on the room temperature performance of TlBr detectors with engineered electrode-TlBr interfaces and three novel metal-oxide electrodes, indium tin dioxide (ITO), titanium dioxide (TiO_2_) and tin dioxide (SnO_2_). Along with excellent adhesion to the TlBr surface (similar to Tl-metal^19^), these non-metallic electrodes demonstrated none to minimal chemical reactivity with the Br-ions, resulting in stable, low-noise detection performance of both planar and pixelated detectors. In addition to the stable performance of planar detectors for more than 4000 hours and their low-noise characteristics, we also report on the continuous performance of a pixelated TlBr detector operated under high unidirectional electric field of 2000 V/cm for more than 2 months (a total of 2880 ^137^Cs response spectra that were collected every half hour).

## Results and Discussion

The TlBr crystals used in this study were grown from in-house purified starting materials^8^. Several large-area planar and pixelated TlBr detectors were fabricated for deposition of the non-metallic electrodes. The non-metallic electrodes of the planar TlBr detectors had an area of 1 to 1.5 cm^2^. For pixelated detectors, 121 pixels with 1.6 mm pitch were deposited on 6-mm thick 20 mm × 20 mm devices.

### Electrode-semiconductor interface

**Figure 1:**
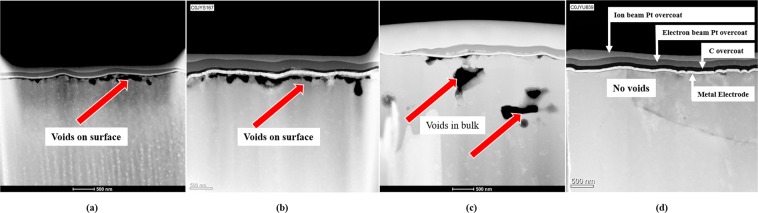
TEM images (500 nm scale shown) showing the presence of voids at the metal-semiconductor interfaces for TlBr detectors prepared using the following techniques: (**a**) Br-etched and (**b**) Cl-etched TlBr surface. (**c**) shows Br-etched TlBr surface after 2-years of bias switching at 1000 V/cm showing voids in the bulk. (**d**) shows void-free interface prepared by a modified process reported in this study. The layers above the voids include: the electrode metal (Pt) layer, and carbon (C) and Pt layers deposited for the TEM imaging.

As TlBr is a soft material (Knoop hardness 11.9 with a 500 g indenter)^20^ and has a chemically reactive surface, surface preparation of TlBr devices prior to electrode deposition needs special considerations. Using focused ion beam transmission electron microscopy (FIB-TEM), we investigated the influence of surface preparation techniques on the formation of defects at the electrode-semiconductor interfaces. The widely-used two step mechanical polishing and chemical etching process with Br and Cl^6,8,9,15,17,21–23^, results in significant number of voids at the electrode-semiconductor interface, Fig. 1(a–c). The TEM images in Fig. 1(a,b) show voids with dimensions of the order of tens of nm distributed across the entire TlBr-Pt interface for Br- and Cl-etched surfaces. These images were obtained before the devices were subjected to ionizing radiation and electric field for charge collection. Figure 1(c) shows a Br-etched sample with a Pt electrode after two years of continuous irradiation by a ^137^Cs source and operation under an alternating applied field of 1000 V/cm at a bias switching frequency of 8-hours. In addition to the voids at the TlBr-Pt interface, large voids within the bulk are observed. As the control sister device shows no such voids in the bulk (Fig. 1(a)), it can be argued that these voids were generated as result of the device operation. Figure 1(d) shows the TEM images of a completely void-free TlBr-Pt interface device fabricated with a modified chemo-mechanical process as described under the methods section later in the paper.

**Figure 2:**
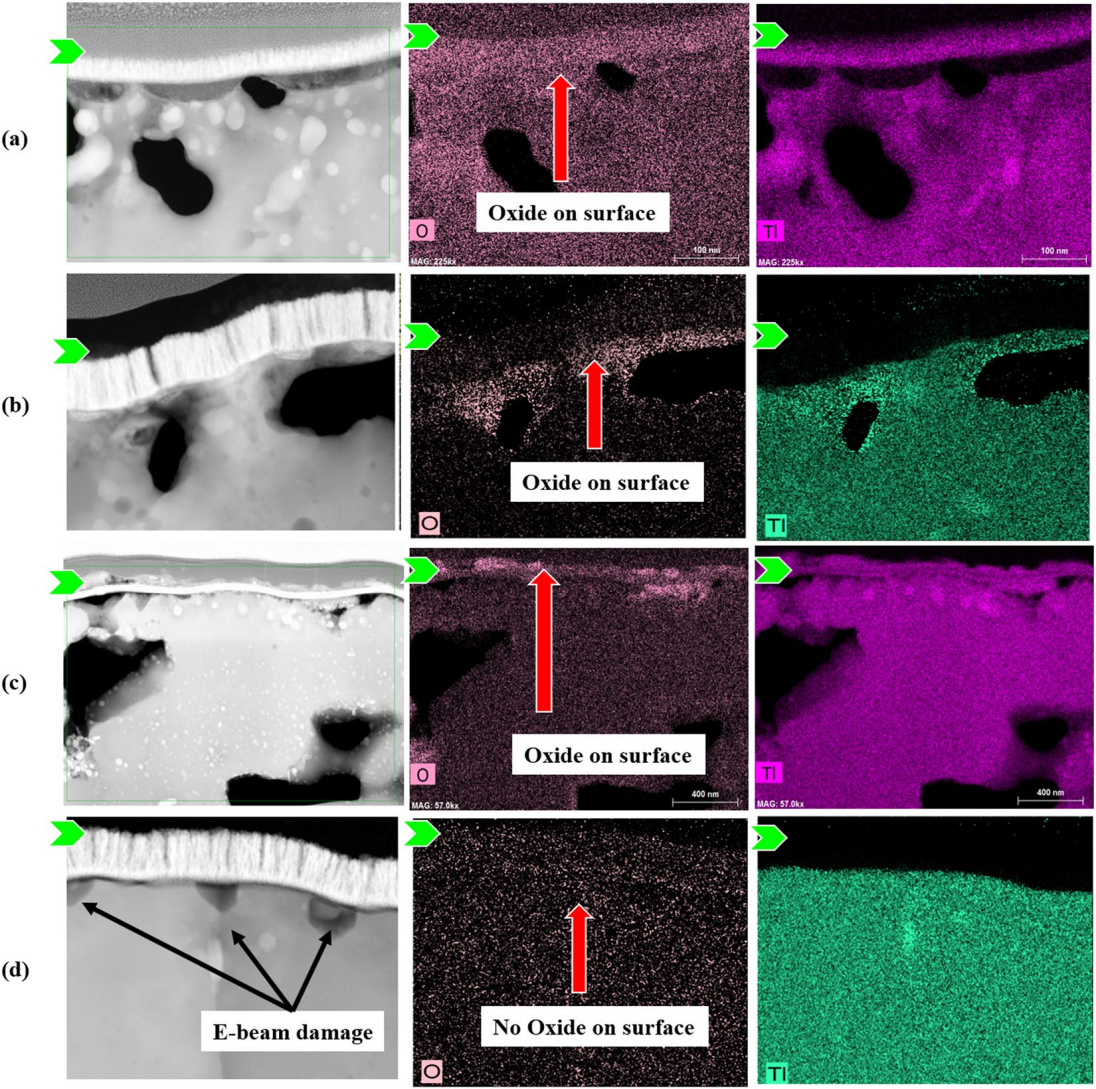
EDS images showing oxygen and thallium concentration variations along the metal-semiconductor interfaces for TlBr detectors prepared with: (**a**) Br-etched and (**b**) Cl-etched TlBr surface, (**c**) Br-etched TlBr surface after 2-years of bias switching at 1000 V/cm, and (**d**) void-free interface prepared by a modified process reported in this study. The dark spots in (**d**) are from E-beam damage during measurement and are not an indicator of the interface quality. Also, the bright spots in the other images are not indicative of any crystalline defect but appeared while the crystal was under high energy E-beam during the experiment. The green arrows indicate top of the metal electrode in the respective figures.

Energy dispersive spectroscopy (EDS) of the samples of Fig. 1(a–c) shows a substantial presence of thallium oxide on the TlBr-side of the TlBr-Pt interface, Fig. 2(a–c). The surface of these samples were prepared in air which explains the oxidation of thallium on the surface. It is reasonable to suggest that if surface preparation were conducted in an inert-gas environment, a thallium-rich layer would have existed prior to metallization in the Br- and Cl-etched samples. In contrast, the sample prepared by the modified procedure shows the absence of thallium-oxide at the TlBr-Pt interface, Fig. 2(d), even though the TlBr surface was prepared in air. The non-uniform spatial distribution of thallium oxide in samples processed by the unmodified procedure can be expected to have a deleterious effect on charge collection and spectral performance of the devices. Elimination of the thallium oxide layer by the modified process promises to reduce device-to-device performance variability, as well as the device performance over time. Results of this study are directly transferable to all the Br-based heavy-metal halide semiconductors including the new generation of perovskite semiconductors such as CsPbBr_3_.

### Novel electrode materials

**Figure 3:**
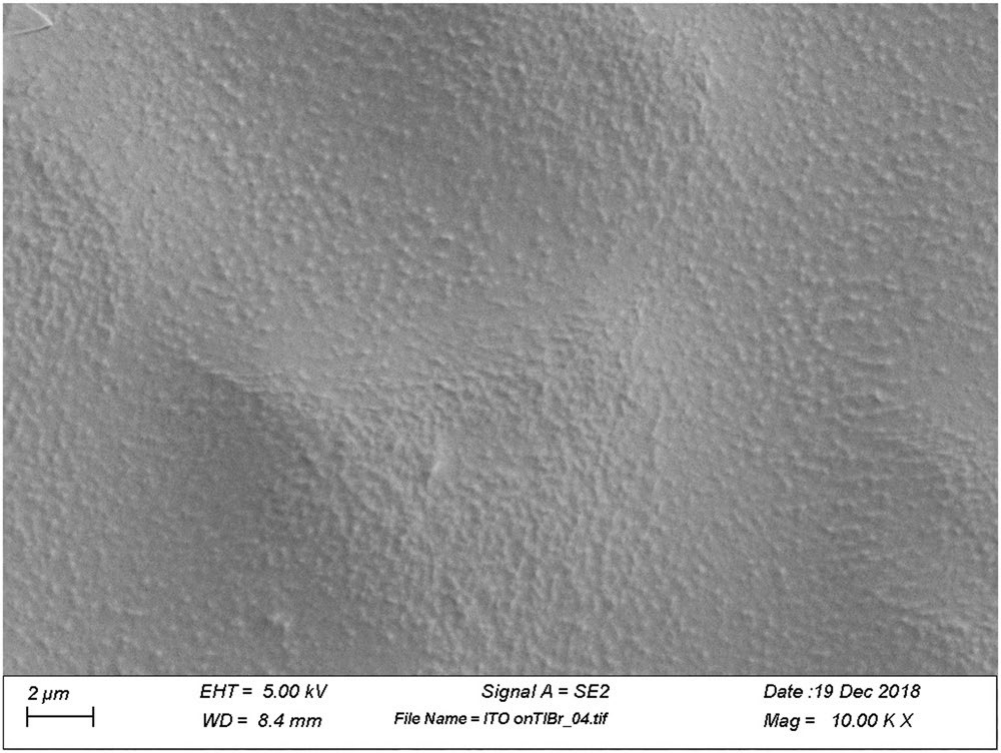
10kX magnification SEM image of 2-nm thick ITO electrode on TlBr. A 2-nm thick indium metal layer was deposited on the non-metallic layers to ensure good interconnection between the bonding wire and the electrode. The spectroscopic charge collection and data acquisition was accomplished using a standard Ortec nuclear pulse processing train and Maestro software The charge collection data shown below were obtained under unidirectional biasing of the device and at room temperature (varying between 10–25 °C); the data was not digitally enhanced, nor corrected for room temperature variations. For control experiments, TlBr detectors were produced with the modified surface preparation process using Pt as the electrode. The control devices were fabricated from TlBr samples from boule positions adjacent to the samples used for measurements reported below.

Devices with several new electrode materials on void-free TlBr surfaces were fabricated and tested. ITO, TiO_2_, and SnO_2_ were deposited by the physical vapor deposition (PVD) technique. There are three main reasons for choosing this technique over other thin film deposition techniques: (1) low processing temperature, (2) uniform layer deposition for low thicknesses films, and, (3) high throughput. As TlBr is a low melting temperature material, the electrode deposition technique is limited to room temperature substrate operation. It was observed that even a slight elevation in the substrate (TlBr) temperature resulted in non-uniform and deteriorated electrodes. In order to keep the temperature of the substrate below 25 °C, an optimum distance was identified between the high-temperature PVD source and the substrate. A scanning electron microscope (SEM) image of uniformly-deposited ITO film on a TlBr substrate is shown in Fig. 3. The observed undulations are related to the preparation of the underlying TlBr surface.

### Current-voltage characteristics

**Figure 4:**
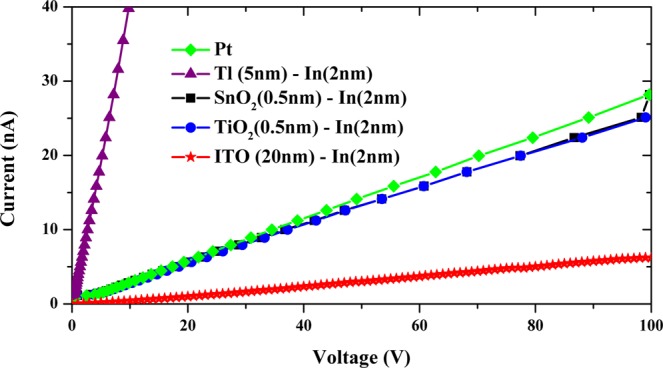
Current-Voltage characteristics of several metallic and non-metallic electrodes on planar TlBr detectors.

**Figure 5:**
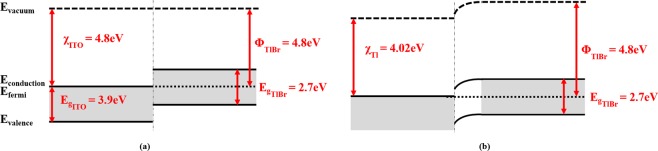
(**a**) Band energy diagram of (**a**) ITO and (**b**) Tl electrodes with TlBr at thermal equilibrium. An estimated value of 6.1 eV was used as the ionization potential of TlBr^24^.

Figure 4 shows the current-voltage characteristics of TlBr devices with metallic and non-metallic electrodes. Table 1 summarizes the average resistivity values from 4 sister devices. The resistivity of the TlBr detector with ITO-films is the highest and with Tl-metal electrode is the lowest. The band energy diagram of the metal-semiconductor interface for ITO (non-metal electrode) and Tl (metal electrode) cases are shown Fig. 5. The respective vacuum, conduction, valence and fermi levels are shown. The electron affinity (χ) of the ITO and Tl electrodes are 4.8 eV and 4.02 eV respectively. As ITO is an n-type degenerate semiconductor, the Fermi level for this contact approaches the conduction band, thereby equalizing the χ and work function (Φ) values. The Φ-value of TlBr was estimated from the published values of ionization potential and the bandgap of TlBr^20,24^. From the current-voltage characteristics, it is clear that the TlBr material used for this study is semi-insulating and self-compensated. Therefore, the Fermi energy level is situated near the midgap region, which results in the Φ value of 4.8 eV. Hence, for the cases mentioned here: χ_ITO_ ≈ Φ_TlBr_ > χ_Tl_. When ITO comes in contact with TlBr, majority charge carriers should flow in their appropriate directions without facing any hindering potential. Ideally, both ITO and Tl are ohmic contacts, which is supported by the current-voltage characteristic curves. The state density at the interface and the stoichiometry of the ITO layer determines the current flow through the interface indicating the differences between the ITO and electrode-metal leakage current values. The lower leakage current with ITO allows for application of higher E-fields across the detectors for better charge-collection performance.

**Table 1:** Resistivity of sister TlBr devices with metallic and non-metallic electrodes under dark conditions.

Electrode type	Resistivity (Ohm-cm)
Pt	4.4 × 10^10^
Tl	3.5 × 10^9^
SnO_2_	5 × 10^10^
TiO_2_	4.9 × 10^10^
ITO	2.4 × 10^11^

The devices with ITO films show highest resistivity whereas Tl-films show the lowest.

### Detector noise

Results presented in this section are extracted from measurements on long-term polarization of TlBr detectors collected over a course of several months. The preamplifier output (V_b_) from the planar detectors were recorded for 24 hours after operation under unidirectional biasing configuration (the detectors were fabricated from similar locations in the crystal boule). The preamplifier baseline voltage (V_b_) fluctuation statistics are given in Table 2. The standard deviation in the baseline response of detectors with non-metallic electrodes under unidirectional bias with electric fields as high as 2500 V/cm is lower than the values for the detector with Pt electrodes operated at substantially lower field of 800 V/cm (7.7 × 10^−4^ V for ITO vs 3.97 × 10^−3^ V for Pt).

**Table 2:** Preamplifier baseline voltage (V_b_) fluctuation statistics (averaged over N_total_ = 25k data points) for TlBr devices with different electrode materials continuously operated under an electric field and ^137^Cs irradiation.

Electrode type	Electric field (V/cm)	Bias type	Days under bias	Mean V_b_	Standard Deviation of V_b_	Variance in V_b_	Mean absolute deviation of V_b_
ITO	2500	Unidirectional	90	−0.1220	7.7458E-4	5.9998E-7	4.4875E-4
SnO_2_	1000	Unidirectional	90	−0.1210	8.2352E-4	6.7818E-7	5.1030E-4
TiO_2_	1000	Unidirectional	90	−0.1219	6.1979E-4	3.8414E-7	3.7207E-4
Pt	800	Unidirectional	45	−0.1215	39.734E-4	1.573E-5	8.3586E-4

The ^137^Cs sources were removed during the collection of detector noise statistics. The device structures include In/metal-oxide anode and cathode. For ITO, high electric field was used to accelerate detector ageing. The performance of detectors with Pt-electrode varies significantly from detector-to-detector, and statistics from a representative dataset is shown in this table.

**Figure 6:**
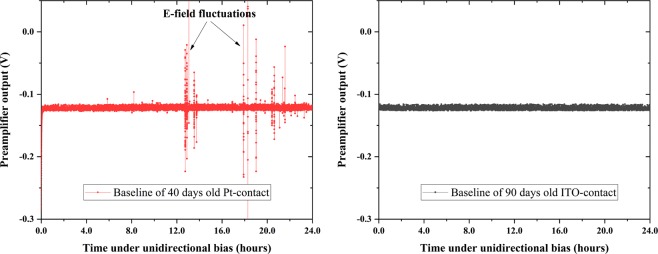
Comparison of electric field stability of two TlBr devices with Pt and ITO electrodes under unidirectional bias. Prior to these measurements, the Pt- and ITO-electrode devices had been under unidirectional bias of 1000 V/cm and 2500 V/cm for 40 and 90 days, respectively. The raw preamplifier signals were digitized and recorded using a low noise microcontroller-based system.

The non-metallic electrodes are the only optimum solution for low noise detectors operated under unidirectional bias. The lower detector noise results in higher signal-to-noise ratio and stable performance over longer periods of time. In addition to the baseline noise, the non-metallic electrodes eliminate baseline instabilities which appear randomly and last for a few minutes. The genesis of these stabilities is not well understood, but it is reasonable to assume that they emerge from sporadic fluctuations of the electric field. Figure 6 shows the comparison of baseline instabilities between TlBr detectors with Pt and ITO electrodes, under unidirectional bias. Prior to these measurements, the Pt-electrode device was biased for 40 days at 1000 V/cm and the ITO-electrode device was biased for 90 days at 2500 V/cm. Results shown in Fig. 6 were taken continuously over 24 hours in absence of any radiation source. Whereas the Pt detector demonstrates substantial baseline fluctuations, the non-metallic detector is completely stable.

### Spectroscopic performance

**Figure 7:**
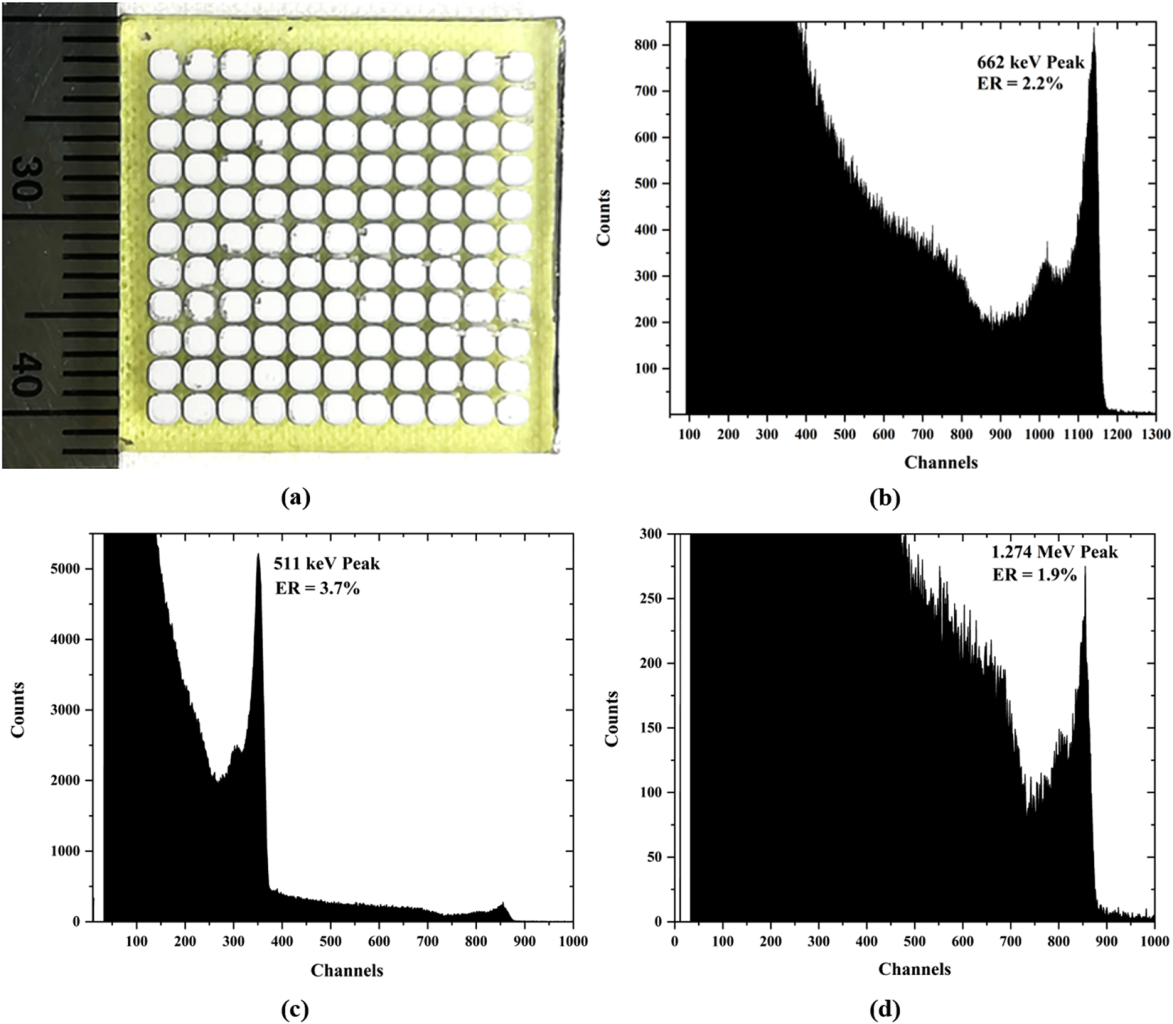
(**a**) 20 mm × 20 mm × 6 mm Pixelated TlBr detectors with 1.6mm pitch ITO/In pixels; (**b**) uncorrected room-temperature ^137^Cs radiation source response of the center 2 × 2 pixels showing energy resolution of 2.2% for 662 keV gamma line; (**c**) uncorrected room-temperature ^22^Na radiation source response showing 511 keV annihilation peak and 1.274 MeV gamma line (magnified in 7 (**d**)). The Tl-escape peaks associated with each of the energies are clearly visible.

The room-temperature spectroscopic properties of the TlBr devices with ITO electrodes (with an overlayer of In) were measured using several large (20 mm × 20 mm × 6 mm) pixelated devices with 121 pixels with 1.6 mm pixel pitch, Fig. 7(a). The uncorrected radiation response of the central 2 × 2 pixels (with the outer pixels connected to ground) to ^137^Cs and ^22^Na gamma sources is shown in Fig. 7(b–d). The energy resolution values for these detectors are comparable to the detectors fabricated using metallic electrodes^21^. These spectra are collected using analog pulse processing electronics without application of any digital signal correction algorithms.

### Long-term performance

**Figure 8:**
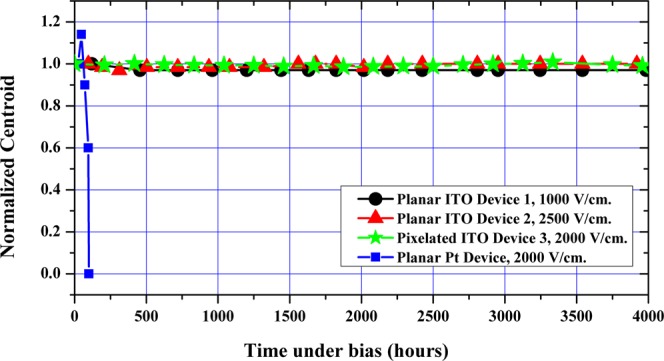
Variations in normalized 662 keV centroid positions of various TlBr detectors over time. Note the superior performance of the non-metallic electrodes in comparison with metallic electrodes for unidirectionally-biased devices with accelerated ageing conditions (i.e. with higher electric fields). The changes in the 59.5 keV peak centroids for unipolar biased device with several surface conditions under continuous bias and ^241^Am irradiation can be seen in the ref.^15^.

Figure 8 shows the performance of several TlBr devices under unidirectional bias for more than 4000 hours. It shows the time history of the normalized 662 keV centroid positions of planar and pixelated detectors with non-metallic and Pt electrodes. The Pt-electrode detector shown here was fabricated using the unmodified surface preparation procedure. These planar detectors were operated at 1000 V/cm and 2500 V/cm under continuous gamma irradiation by 10µCi ^137^Cs sources. The short lifetime of the device with Pt electrode is consistent with other reported data for metal electrodes which range from 100 to 2400 hours (5 to 100 days)^5,13^. In general, the metal-electrode TlBr devices show a high degree of irreproducibility and significant baseline noise, as shown in the previous section. In contrast, the devices with ITO electrode exhibit extreme stability over 4000 hours of continuous operation, even at high electric fields of 2500 V/cm.

**Figure 9:**
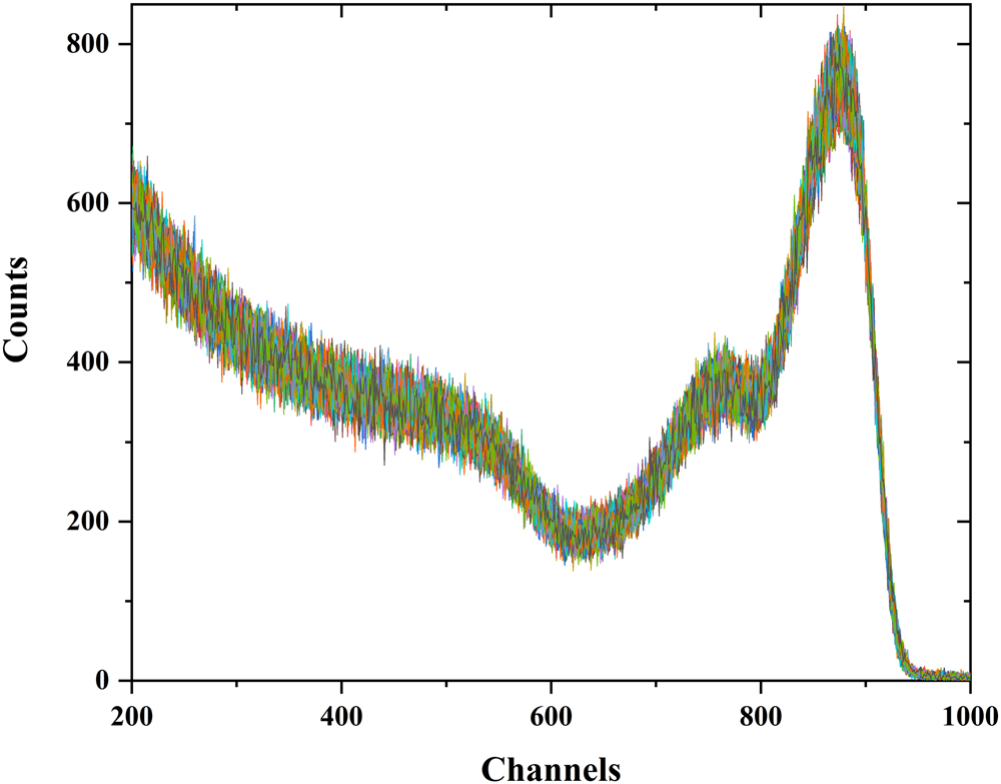
Superimposed 2880 uncorrected spectra of a pixelated TlBr detector with non-metallic electrodes and ^137^Cs radiation source recorded every 30 minutes for two months (1440 hours). The ambient temperature and humidity variations (~10 to 25 °C and 2 to 79%RH) have likely influenced the observed spread of 662 keV peak characteristics^25^. The spread in the 662 keV centroid throughout this time is limited to ±1%.

The continuous performance of a pixelated TlBr detector with ITO electrodes collected over 2 months (1440 hours) is shown in Fig. 9. A total of 2880 ^137^Cs response spectra were collected at 1500 second intervals, using an analog pulse processing chain. The device stability is evident by the significantly low variation in the channels and counts. The 662 keV centroid deviation is about ±1%, a part of which is related to fluctuations in ambient temperature and humidity (~10 to 25 °C and 2 to 79%RH). Such minor deviations can be compensated through standard instrument recalibration protocols, say after every 1000 hours of operation. Then, the channel drift becomes less than 0.1%.

**Figure 10:**
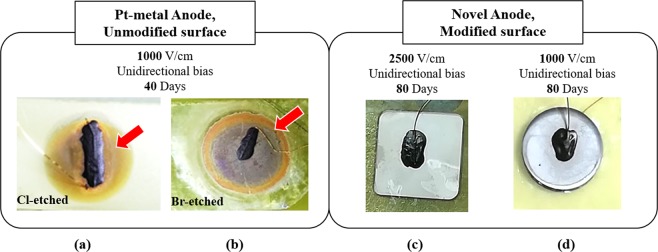
(**a**,**b**) Pt anodes after 40 days of unidirectional bias deposited on Br- and Cl-etched TlBr surfaces. The orange “halo” (shown by the red arrows) and the dark green/violet hue of the electrode indicate the metal-Br reaction due to electromigration of Br-ions. (**c**,**d**) Non-metallic anodes (ITO and TiO_2_) with In-overcoat after unidirectional biasing for 80 days under higher electric fields. There is no visible “halo” or discoloration of the electrodes. The black mass on the electrodes is the carbon glue used for attaching the preamplifier input to the electrode via a gold wire. The variations in the surface texture of the non-metallic electrodes are a result of deposition non-uniformities and were present since the beginning of the ageing experiment.

Figure 10 shows the physical conditions of the Pt, ITO and TiO_2_ anodes from TlBr devices operated under unidirectional bias. The Pt electrodes exhibit an orange “halo” indicative of significant reaction with bromine associated with the electromigration of Br-ions during device operation. In contrast, there are no visual indications of any degradation of the non-metallic contacts.

Ionic polarization in the halide detectors can be categorized into short and long-term processes^18^. The rate of the short-term process is determined by the magnitude and direction of the applied electric field and that of the latter is controlled by availability of free Br-ions near the metal electrode. In a previous study, we have shown that bias switching i.e. periodic changes in the bias polarity of the detector, significantly minimizes the short and long-term effects of ionic polarization^18^. Based on those results and those of this study, the following explanation of the effect of non-reactive electrodes on device performance can be made. Under unidirectional biasing, the continuous Br-ion migration through V_Br_^+^ sites are highly pronounced and increases over time^8^. This higher diffusion flux results in the short-term polarization of the device, alongside making more Br-ions available near the anode-TlBr interface. When the anode is metallic, this stream of Br- ions reacts with the anode creating a virtual sink for Br-ions. In contrast, due to the non-reactivity of non-metallic electrodes with Br- ions, there is no “sink” for the free Br^−^ ions. This in turn minimizes the vacancy concentration in the region close to the contact which leads to unavailability of migration paths for the Br-ions under the electric field. The increase in the concentration of Br- ions at the electrode-TlBr interface sets up a gradient in the concentration of Br- ions which, in turn, would lead to a back-diffusion of the ions due to Fickian diffusion. Thus, the electromigration of Br-ions is counterbalanced by Fickian diffusion, resulting in an equilibrium concentration of Br-ions occupying the available vacancies in the electrode-TlBr interfacial region. According to Fick’s first law, the diffusion flux J_f_ is proportional to the gradient of the Br-ion concentration φ,

$${J}_{f}=-\,D{\nabla }\phi $$

In the above, D is the phenomenological diffusion coefficient. At equilibrium the Fickian diffusion flux is balanced by the electromigration flux J_e_:

$${J}_{e}=\frac{ne}{kT}Z\ast E$$

where, n is the mass density of migrating elements (primarily Br^−^), e is the charge of electron, k is Boltzmann constant, T is the device temperature, Z* is the effective charge number for the migrating ions, and E is the electric field. The equilibrium Br- ion gradient is then:

$$-\,{\nabla }\phi =\frac{ne}{DkT}Z\ast E$$

The equilibrium concentration of Br-ions is disturbed by external changes in the device temperature and operational changes such as the electric field strength. As long as these parameters are kept constant, the effect of Br- ions on the devices with non-metallic electrodes should remain stable over long period of time.

**Figure 11:**
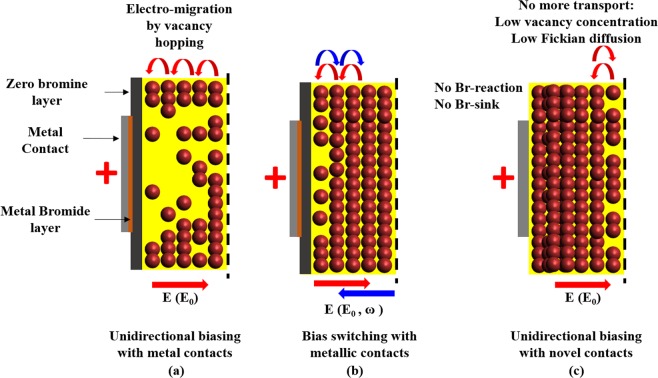
(**a**) Metal-TlBr interfacial region in a typical TlBr device under unidirectional and (**b**) bias switched configurations^18^. The brown region at the metal semiconductor interface depicts the layer of metal bromide that forms when free Br^−^ ions chemically reacts with the metal. (**c**) In the case of non-metallic contacts, there is no reaction between the metal and Br-ions which eliminates the Br-sink and provides the detector with a steady state for stable long-term operation. Partially reprinted from ref.^18^ with permission.

Figure 11 shows three ion-transport scenarios for TlBr detectors with unidirectional biasing using metallic and non-metallic contacts as well as bias-switching.

In conclusion, to counteract the deleterious effects of electromigration of Br ions and their subsequent reaction with the contact metal in unidirectionally biased halide semiconductor devices, we have successfully implemented two solutions. First, a new approach to the preparation of the semiconductor surface prior to application of the contact which essentially eliminates voids at the contact-semiconductor interface observed in otherwise prepared semiconductor surface. Second, novel bandgap-tuned non-metallic electrodes that do not react with Br ions during device operation. TlBr detectors fabricated by the techniques described in this paper demonstrate highly resistive ohmic interfaces, no baseline noise, extremely stable operation over long periods of time, and a long room temperature lifetime. These attributes have not been achievable in TlBr detectors with metallic contacts and semiconductor surfaces etched by bromine- or chlorine-based chemicals. Results of this work suggest that the major obstacle to the widespread adoption of TlBr have been overcome. Solutions discussed in this paper may be also extended to other halide semiconductor detectors.

## Methods

### Crystal growth and device fabrication

TlBr detector grade crystals (1.5-inches in diameter) used in this study were grown by the Travelling Molten Zone technique (TMZ) using 5 N purity anhydrous TlBr beads from EMD Performance Materials^5,6,14^. The beads were melted, and zone refined at 5 cm/h. After 100 zone refining passes, single crystal growth was achieved by reducing the translation rate to 1 mm/h. Stoichiometry of the molten phase was controlled by using a mixture of HBr and an inert gas in the growth atmosphere. The TlBr crystal was cut into detector-size pieces with a diamond wire saw. The crystals were then grinded using SiC paper and polished chemo-mechanically on a soft pad. For the modified chemo-mechanical polishing, the TlBr devices were grinded and polished using alcohol before a quick-dip (less than 5 seconds) in the etching solution of HBr-H_2_O_2_ in 50–90wt% water. Before placing the electrodes, the polished surfaces were cleaned using Methanol and plasma, and dried using Argon. During the PVD electrode deposition procedure, the starting materials (ITO, TiO_2_, SnO_2_) were placed on a heat source and a mixture of argon and oxygen was flown through the system while keeping the pressure close to 10^−3^ Torr. An optimum distance was maintained between the high-temperature source and the TlBr detector while maintaining a deposition rate of 5 Å/sec. A metal shadow mask was used for creating the pixel pattern on the TlBr detectors.

### Imaging studies

The TEM-ready samples were prepared using the *in-situ* FIB lift out technique on a FEI Dual Beam FIB/SEM. The samples were capped with sputtered C and e-Pt/I-Pt prior to milling. The TEM lamella thickness was ~100 nm. The samples were imaged with a FEI Tecnai TF-20 FEG/TEM operated at 200 kV in bright-field (BF) TEM mode, high-resolution (HR) TEM mode, and high-angle annular dark-field (HAADF) STEM mode. The STEM probe size was 1–2 nm nominal diameter. EDS spectra were acquired on Oxford INCA, Bruker Quantax EDS system.

## Data Availability

The data that support the findings of this study are available from the corresponding author upon reasonable request.

## References

[1] He Y (2018). High spectral resolution of gamma-rays at room temperature by perovskite CsPbBr_3_ single crystals. Nat. Comm..

[2] Mizusaki J, Arai K, Fueki K (1983). Ionic conduction of the perovskite-type halides. Solid State Ionics.

[3] Eames C (2015). Ionic transport in hybrid lead iodide perovskite solar cells. Nat. Comm..

[4] Roy UN (2019). Growth of CdMnTe free of large Te inclusions using the vertical Bridgman technique. J. Cryst. Growth..

[5] McMaster, W. H., Grande, N. K. D., Malett, J. H. & Hubbel, J. H. Compilation of X-ray Cross Sections, Lawrence Radiation Laboratory, (UCRL-50174- SEC. 2-R1) (1969).

[6] Hitomi K, Onodera T, Kim S, Shoji T, Ishii K (2014). Characterization of pixelated TlBr detectors with Tl electrodes. Nucl. Inst. Meth. Phys. Res. A..

[7] Hitomi K, Kikuchi Y, Shoji T, Ishii K (2009). Polarization Phenomena in TlBr Detectors. IEEE Trans. Nucl. Sci..

[8] Datta A, Becla P, Motakef S (2015). Visualization of TlBr ionic transport mechanism by the Accelerated Device Degradation technique. Nucl. Inst. Meth. Phys. Res. A..

[9] Datta A, Motakef S (2015). Cathode Degradation in Thallium Bromide Devices. IEEE Trans. Nucl. Sci..

[10] Costa F, Mesquita C, Hamada M (2009). Temperature Dependence in the Long-Term Stability of the TlBr Detector. IEEE Trans. Nucl. Sci..

[11] Kozorezov A (2010). Polarization effects in thallium bromide x-ray detectors. J. Appl. Phys..

[12] Leao CR, Lordi V (2013). Ionic current and polarization effect in TlBr. Phys. Rev. B.

[13] Holzer A, Scheiber M (1980). Reduction of polarization in mercuric iodide nuclear radiation detectors. IEEE Trans. Nucl. Sci..

[14] Datta. A, Motakef S (2015). Characterization of Stress in Thallium Bromide Devices. IEEE Trans. Nucl. Sci..

[15] Conway AM (2013). Fabrication Methodology of Enhanced Stability Room Temperature TlBr Gamma Detectors. IEEE Trans. Nucl. Sci..

[16] Donmez B, He Z, Kim H, Cirignano LJ, Shah K (2010). The stability of TlBr detectors at low temperature. Nucl. Inst. Meth. Phys. Res. A..

[17] Datta A, Moed D, Becla P, Overholt M, Motakef S (2016). Advances in crystal growth, device fabrication and characterization of thallium bromide detectors for room temperature applications. J. Crys, Growth.

[18] Datta A, Fiala J, Becla P, Motakef S (2017). Stable room-temperature thallium bromide semiconductor radiation detectors. Appl. Phys. Lett. Mater..

[19] Datta A, Becla P, Motakef S (2018). Thallium Bromide Semiconductor Radiation Detectors with Thallium Contacts. IEEE Trans. Nucl. Sci..

[20] Datta A, Becla P, Motakef S (2018). Large area thallium bromide semiconductor radiation detectors with thallium contacts. Proc. SPIE.

[21] Hitomi K, Onodera T, Shoji T (2007). Influence of zone purification process on TlBr crystals for radiation detector fabrication. Nucl. Inst. Meth. Phys. Res. A..

[22] Zhiping Z, Yongtao Y, Dongxian Z, Shuping G, Qiuyun F (2014). Research on annealing and properties of TlBr crystals for radiation detector use. Nucl. Inst. Meth. Phys. Res. A..

[23] https://www.crystran.co.uk/optical-materials/thallium-bromide-tlbr (2019).

[24] Lordi, V. Background Information for Independent Review Team: Lifecycle Plan and FY14 Quarterly Reports, LLNL-TR-663374, (2014).

[25] Onodera T, Hitomi K, Shoji T (2007). Temperature Dependence of Spectroscopic Performance of Thallium Bromide X- and Gamma-Ray Detectors. IEEE Trans. Nucl. Sci..

